# Serological biomarker testing helps avoiding unnecessary endoscopies in obese patients before bariatric surgery

**DOI:** 10.1186/s40608-018-0185-5

**Published:** 2018-02-20

**Authors:** Jaanus Suumann, Toomas Sillakivi, Živile Riispere, Kari Syrjänen, Pentti Sipponen, Ülle Kirsimägi, Ants Peetsalu

**Affiliations:** 1 Department of Surgery, University of Tartu, Tartu, Estonia; 2 Department of Pathology, University of Tartu, Tartu, Estonia; 3 Department of Clinical Research, Biohit Oyj, Helsinki, Finland; 4 Patolab Oy, Espoo, Finland

**Keywords:** Morbid obesity, Bariatric surgery, Gastroscopy, Serological biomarkers, Test accuracy, Histological evaluation, Sydney system (USS), GastroPanel

## Abstract

**Background:**

To assess the value of serological biomarker testing as a substitute for esophagogastroduodenoscopy (EGDS) in pre-operative assessment of patients referred for bariatric surgery.

**Methods:**

Sixty-five obese patients with a mean age of 43 years (range: 21–65) and a mean body mass index (BMI) of 44 (range: 36–59) were studied. The patients were tested with a four-biomarker panel: pepsinogen I and II, gastrin-17 (basal and stimulated), and *Helicobacter pylori* (HP) antibodies (GastroPanel®, Biohit Oyj, Finland). On the basis of the biomarker test, the patients were classified into the HS (healthy stomach) group (*n* = 22) with the normal biomarker profile and the NHS (non-healthy stomach) group (*n* = 43). The classification of patients into HS and NHS was evaluated against the gold standard, i.e. EGDS with biopsies.

**Results:**

The concordance (Cohen’s kappa) between the biomarker test and gastric histology was 0.68; 95% CI 0.504–0.854, with an overall agreement of 84.6% (95% CI 73.9–91.4%). In the NHS group, all 43 patients had biopsy-confirmed chronic gastritis: 39 non-atrophic HP-gastritis, 4 atrophic antrum gastritis (AGA) of moderate severity.

In the HS group only 6 patients had mild superficial H.pylori negative gastritis. Of the 22 HS subjects with the normal biomarker profile, 20 (31% of all 65) had no complaints either, while the remaining two had reflux symptoms with esophagitis. In the NHS group 10 patients had esophagitis and 8 had also reflux symptoms.

**Conclusions:**

The normal biomarker profile is an excellent surrogate for healthy stomach, implicating that pre-operative EGDS could have been avoided in 31% of our asymptomatic bariatric surgery patients who had the normal biomarker profile.

## Background

Currently, overweight and obesity are a major priority in global healthcare, affecting over 600 million adults and the figures have more than doubled since 1980 [1–4]. Obesity is an independent risk factor for a variety of chronic diseases, including hiatal hernia, gastroesophageal reflux disease (GERD), erosive esophagitis, Barret’s esophagus and esophageal adenocarcinoma [5–10].

During the past three decades, bariatric surgery has gained an increasingly important role in the management of the most severe cases of obesity [4]. In the current clinical practice, esophagogastroduodenoscopy (EGDS) has been the gold standard in the preoperative investigation of all patients referred for bariatric surgery, but its routine use in asymptomatic patients has been questioned [10–14]. The opponents argue that preoperative EGDS findings rarely change surgical management [15–17]. However, there is unanimous agreement that all bariatric patients with upper gastrointestinal (UGI) complaints should undergo preoperative EGDS [17, 18]. This is because bariatric operation includes surgery of the stomach, which makes accurate preoperative assessment important and contributes to the patient set-up and operation type. The question remains, however, whether systematic EGDS could be replaced by another (non-invasive) diagnostic tools in this setting.

During the past decade, the use of serological biomarker testing has gained increasing popularity as a non-invasive diagnostic tool for dyspeptic patients and asymptomatic subjects to diagnose both functional disorders and gastric diseases, including HP infection and atrophic gastritis (mucosal atrophy) (AG) [19]. The latest innovation in this technology represents a panel of 4 stomach-specific biomarkers (Pepsinogen I and II, Gastrin-17 and HP antibody) known as the GastroPanel® test (Biohit, Oyj, Finland), which distinguishes between 8 diagnostic marker profiles [19]. Apart from the perfectly normal profile, three others represent purely functional disorders (in acid output) while the remaining four indicate morphological abnormalities (HP and AG). With its very high negative predictive value (> 95%), the normal marker profile excludes any significant gastric pathology with high probability. On the other hand, accurate diagnosis of the gastritis phenotype and topography (antrum or corpus) are important because of their different risks for gastric cancer and/or peptic ulcer [19]. Today the validity for GastroPanel to diagnose and delineate the healthy stomach and *H. pylori* gastritis with or without atrophy has already been confirmed in many independent clinical investigations against the gold standard (endoscopy with endoscopic histology); this issue has been adressed in at least two systematic reviews [20, 21].

The serological biomarker test is a non-invasive diagnostic tool which is substantially less expensive than EGDS (e.g. in Finland the cost of GP is about 125 EUR vs 600 EUR for gastroscopy with histopathological evaluation). On the basis of the GastroPanel test, it is straightforward to select patients for whom gastroscopy is mandatory, i.e. those with AG (antrum, corpus or both). In contrast, gastroscopy is not needed for patients who present with HP-infection alone (with no AG); conventional HP eradication is sufficient management [19]. Similarly, no additional information can be obtained by means of gastroscopy in those patients who have a normal marker profile, despite the fact that minor lesions (e.g. non-specific inflammation, mucosal irritation or micro-erosion) are not excluded by the normal marker profile.

Given the existing divergent opinions on the role of EGDS in the management algorithm of obese patients referred for bariatric surgery [10–18], we designed the present study to elucidate the role of non-invasive biomarker testing in the pre-operative evaluation of these patients. Using gastroscopy and biopsies as the gold standard, all patients were tested with GastroPanel® to establish the concordance between these two techniques. One of the aims was to evaluate the reliability of the normal marker profile (also called “healthy stomach”, HS) to predict a biopsy-confirmed healthy gastric mucosa. This should have direct bearing with the key clinical question: how many EGDS examinations can be avoided by systematic biomarker testing of obese patients before bariatric surgery?

## Methods

This study is a part of an ongoing prospective cohort study of 65 obese patients who underwent bariatric surgery at Tartu University Hospital. The key patient characteristics are presented in Table 1. The mean age of the patients was 43.1 years (SD 9.1), and the mean BMI was 44.3 (SD 5.1). Of the 65 patients, 44(68%) were women. All patients were eligible for bariatric surgery: i.e., a BMI of > 40 or > 35, with certain obesity-related comorbidities. In accordance with the approved study design, all patients underwent the following pre-operative examinations: serum sampling for biomarker testing, recording the history of upper abdominal complaints (dyspepsia, heartburn), routine EGDS examination with directed biopsies, and their histological evaluation.

**Table 1:** Key characteristics of the patients

Patient characteristics	
Age (years, mean ± SD)	43.1 (9.08)
Females *n*(%)	44 (67.7)
Preoperative weight (kg, mean ± SD)	128.3 (21.5)
Preoperative body mass index (Kg/m^2^, mean ± SD)	44.3 (5.12)
Concomitant diseases (any) *n*(%)	57/65 (87.7)
Type II diabetes *n*(%)	9 (13.8)
Hypertonia, cardiac disease *n*(%)	39 (60)
Obstructive sleep apnea *n*(%)	20 (30.7)
Degenerative joint disease *n*(%)	13 (20)
Hypercholesterolemia *n*(%)	42 (64.6)
Smokers *n*(%)	17 (26.1)

The serological biomarker test (GastroPanel® test, Biohit Oyj, Helsinki, Finland) follows the manufacturer’s instructions, as previously detailed [20]. All samples were properly stored and transported to the service laboratory of Biohit Oyj for analysis.

### GastroPanel interpretation

GastroPanel is an automated ELISA test that measures the plasma levels of the following biomarkers: Pepsinogen I (PgI) and II (PgII), fasting (basal) and stimulated amidated G17 (G17b and G17 s), HP antibodies (HPAb). The manufacturer-validated reference values of the four biomarkers were used: PgI 30-160 μg/l; PgII 3-15 μg/l; PgI/PgII ratio 3–20, G17b 1-7 pmol/l; G17 s 3–30 pmol/l; HPAb<30EIU [22–24]. The results are interpreted, using the special GastroSoft® software, by classifying the results according to the biomarkers levels into one of the five diagnostic categories: 1) normal profile, 2) superficial (HP) gastritis (PgI, PgII, PgI/PgII, G-17 Normal or High; HPAb >30EIU), 3) atrophic gastritis of the antrum (AGA) (PgI, PgI/PgII Normal; G-17b Low; HPAb>30EIU), 4) atrophic gastritis of the corpus (AGC) (PgI, PgI/PgII Low; G-17b High; HPAb <30EIU or >30EIU), or 5) atrophic pan-gastritis (AG of the antrum and corpus) (AGP) (PgI Low, PgII Normal or Low; PgI/PgII Low, G-17b Low; HPAb >30EIU), as detailed elsewhere [19, 23, 25].

### Esophagogastroduodenoscopy (EGDS)

During EGDS, the esophageal, gastric and duodenal mucosa was visually inspected and abnormalities were detected. The degree of esophagitis was evaluated according to the Los Angeles classification (LA) [26]. In every patient, two biopsies from the antrum (2 cm from the pyloric ring) and two biopsies from the corpus were collected. Additional biopsies were taken only when necessary. The histology of the biopsies was evaluated separately for the gastric antrum and corpus, according to the updated Sidney System (USS) classification [24], by 3 independent pathologists (ZR, KS, PS). In the case of discrepant results, the biopsies were re-evaluated by a pathologists’ panel, and the consensus diagnosis was used as the final one. HP-colonization and its abundance were semi-quantitatively estimated, separately in the antral and corpus mucosa, by microscopic counting, as absent, mild, moderate and severe, as described earlier [27].

The classification of patients into the HS and NHS groups according to the Gastropanel results was evaluated against the gold standard, EGDS with biopsies.

### Statistical analysis

For statistical analysis, the patients were divided into 2 groups: HS (healthy stomach) group (=normal GastroPanel profile) and NHS (non-healthy stomach) group (all abnormal profiles). All data were compared across these two groups. All statistical analyses were performed using the Statistica 13 software package. To evaluate the agreement between the gastric histology (USS) and the biomarker results (GastroPanel), regular Cohen’s kappa test (with 95%CI) was used. Continuous variables were presented as mean values with standard deviation (SD) and categorical variables were presented as absolute and relative frequencies. The Chi-square and Fisher’s exact tests were employed to assess differences in the categorical data. The non-parametric Mann-Whitney test was used to assess differences in the biomarker values across the study groups. All *P* values were two-sided, a difference was considered statistically significant at *P* < 0.05.

## Results

Table 2 shows concordance between the GastroPanel test and USS histology (5 categories in both). Using the kappa test, agreement between the two methods was significant: Kappa = 0.68 (95% CI 0.504–0.854), with an overall agreement of 55/65 (84.6%; 95% CI 73.9–91.4%) across 5 diagnostic categories.

**Table 2:** Concordance between the biomarker test results and histological diagnosis

	Gastric mucosa histology (USS classification^a^)						
Diagnostic Categories of the GastroPanel test		Normal	Superficial (HP) Gastritis	Antral atrophy (AGA)	Corpus atrophy (AGC)	Panatrophy (AGP)	Total
	Normal profile	16	6	0	0	0	22
	Superficial (HP) Gastritis	0	38	3	0	0	41
	Antral atrophy	0	0	1	0	0	1
	Corpus atrophy	0	0	0	0	0	0
	Panatrophy	0	1	0	0	0	1
	Total	16	45	4	0	0	65

^a^ The Updated Sydney System (USS) classification of gastritis

Using the two-tier stratification (HS vs. NHS), based on biomarker testing, 22 (34%) of the patients were classified into the HS (normal marker profile) group, and 43 (66%) into the NHS group. The biomarker values in these two patient categories are shown in Table 3. Not unexpectedly, the biomarker values in the two groups were significantly different, all except for the PgI/PgII ratio that showed higher values in the NHS group.

**Table 3:** The biomarker levels in the two categories (HS/NHS) of patients

	Whole Series	Healthy stomach (HS Group) n = 22	Non-healthy stomach (NHS Group) n = 43	(*p*-value)#
^a^G17b pmol/l mean ± SD (range)	3.9 ± 5.0 (0–28.1)	2.05 ± 2.2 (0–9.9)	4.9 ± 5.7 (0–28.1)	0.028
^b^G17 s pmol/l mean ± SD (range)	12.3 ± 10.3 (0–40.9)	8.8 ± 8.4 (0.2–36.1)	14.1 ± 10.8 (0–40.9)	0.048
PG I μg/I mean ± SD (range)	81.8 ± 38.6 (22.7–197.5)	58.2 ± 27.9 (33.0–151.1)	93.9 ± 38.0 (22.7–197.5)	< 0.0001
PG II μg/I mean ± SD (range)	7.2 ± 5.9 (0.3–29.3)	3.37 ± 1.7 (0.3–8.4)	9.2 ± 6.4 (1.4–29.3)	< 0.0001
PG I/PG II mean ± SD (range)	15.5 ± 12.3 (3.3–103.3)	21.6 ± 18.9 (10.7–103.3)	12.4 ± 4.6 (3.2–25.2)	< 0.0001
IgG EIU mean ± SD (range)	64.2 ± 40.8 (2.1–121.1)	13.12 ± 7.7 (2.1–27.3)	90.4 ± 21.1 (38.1–121.1)	< 0.0001

^a^ G-17b, basal gastrin-17

^b^ G-17 s, stimulated gastrin-17

#Mann-Whitney U-Test

The prevalence of HP infection in the entire cohort was 43/65 (66%) in the GastroPanel test and 41/65 (63%) in the histological evaluation of the biopsies. In the HS group (*n* = 22), 6/22 (27%) patients had mild chronic HP-negative gastritis in the antrum or corpus, without intestinal metaplasia or substantial activity. There was no statistical difference in any of the biomarkers between these 6 patients (with minor histological findings) or in the remaining 16 patients in the HS group.

In the NHS group (*n* = 43), all patients had histologically confirmed chronic gastritis: 39 patients had non-atrophic HP- gastritis and 4 had AG. The HP- related chronic gastritis was detected as follows: in the antrum, mild or moderate gastritis in 36 cases and severe in 5 cases; in the corpus, mild or moderate gastritis in 36 cases and severe in 2 cases. Four patients had moderate degree AGA at histological evaluation. The G17b (1.6 pmol) and G-17 s (2.7 pmol/l) were low in the first case and low also in the second case (G-17b 0.16 and G-17 s 1.97 pmol/l (over three-fold increase in G17 s but still below the 3 pmol threshold). In the other two cases of AGA, the G17 levels were normal (3.4/12 and 2.6/9.6 pmol/l, respectively, for G-17b and G-17 s, indicating a normal function of the antral G cells. In none of the cases, atrophy was detected in the gastric corpus mucosa (AGC).

In one patient, classified as a case of panatrophy by GP, the histological evaluation did not confirm the finding, i.e. *H. pylori* related chronic superficial gastritis: antrum- moderate degree chronic gastritis with mild activity, mild degree *H. pylori* infection, no atrophy; corpus- moderate degree chronic gastritis with mild activity, mild degree *H. pylori* infection, no atrophy.

In the HS group, hiatal hernia was endoscopically diagnosed in 4 patients and 2 of them had reflux complaints with LA grade A and grade B esophagitis at EGDS. Three patients in the HS group had erosions at EGDS with a modified Lanza score [28] of 2, 2 and 3, respectively. All these 3 patients with erosions were HP-negative, with PgII levels of 3.8, 3.4 and 3.8 μg/l, i.e., with no increase in this mucosal inflammation marker. They all used multiple medications (cardiac, glucocorticoids etc.) due to comorbidities.

Table 4 summarizes the clinical (endoscopic) findings in the HS and NHS groups. Importantly, 20/65 patients (31%), all from the HS group, had no clinical symptoms or HP-associated gastritis, and their biomarker profile was also normal.

**Table 4:** Endoscopic findings, clinical symptoms in the pre-operative assessment of the HS and NHS groups

	Healthy stomach (HS Group) n = 22	Non-healthy stomach (NHS Group) *n* = 43
Endoscopic findings:		
• Hiatal hernia	4	15
• Esophagitis	4	10
• Erosions/erosive gastritis	3	14
• Duodenal polyp	–	1
Clinical symptoms:		
• Reflux disease (GERD)	2	8

*GERD* gastroesophageal reflux disease

In the NHS group, hiatal hernia was endoscopically confirmed in 15/43 patients and esophagitis in 10/43 patients, while 8 had reflux complaints. Only 2 of the 10 patients with esophagitis had G17b levels below 1 pmol/l (0.75 and 0.16); the remaining 8 had the G-17b levels between 1.6 pmol/l and 13.9 pmol/l. In 4 patients with reflux symptoms, esophagitis was confirmed on EGDS: LA grade A in 3 patients and Grade B in 1 patient. In the NHS group, 14 patients also had erosions /erosive gastritis and 1 patient had incidental duodenal polyp. In the NHS group (with 14 patients having erosions), the mean PgII levels were almost three times as high as those in the HS group (Table 3).

There was no correlation between G17b and esophagitis at EGDS or between G17b levels and GERD complaints irrespective of the fact whether patients belonged to the HS or NHS group (all p = NS).

## Discussion

According to the gastric biomarker test results, our patients were divided into the HS and NHS groups, following previous suggestions [19]. Using gastroscopic biopsies as the gold standard, the concordance between the biomarker testing and histology was substantial, with a kappa value of 0.68 and an overall agreement (across 5 categories) of 84.6%. These values favourably compete with those reported in previous validation studies [19, 23]. This is not unexpected because the biomarker test used (GastroPanel) is based on four biomarkers reflecting the function and structural integrity of the stomach mucosa [19, 22, 23, 25, 29]. Accordingly, Pepsinogen levels and their ratio are decreased in corpus atrophy, accompanied by elevated G-17. The G-17 level also gives indication of gastric acid secretion, being low with high acid output and high when stomach is acid-free (due to PPI treatment or AG). In antrum atrophy, G-17 is low and does not respond to protein stimulation (lack of G-cells). The two main indications of the GastroPanel test are: 1) first-line diagnostic test for dyspeptic complaints, and 2) screening of asymptomatic subjects for gastric cancer risk (HP and AG). Despite the fact that some minor abnormalities are not detected by the normal marker profile, GastroPanel is a test for stomach health, with an excellent longitudinal negative predictive value. On the other hand, abnormal test results implicating AG do predict a significantly increased long-term risk for gastric cancer.

The present study is the first where the utility of this biomarker test in the pre-operative management of bariatric surgery patients was evaluated in a 100% biopsy-confirmed clinical setting. One of the aims was to assess how many gastroscopies could be avoided by using the normal biomarker profile as a surrogate for healthy stomach (HS). The results are encouraging, while implicating that using this approach, the HS and NHS groups can be distinguished with high accuracy. Indeed, the serum levels of all 4 biomarkers were significantly different in the HS and NHS groups, being markedly higher in the latter (Table 3). Most remarkably, the markers of mucosal inflammation (PgII in particular) was almost three times as high in the NHS patients as in the HS patients.

HP is the key causative factor of severe gastric pathology, including peptic ulcer disease and gastric cancer. In the study cohort, the prevalence (66%) of HP in candidates for bariatric operation was significantly higher than that reported in many previous studies (3.4–17%) from Belgium, Finland and the USA [13, 15, 30], but similar to that (53–66%) reported from Greece and Brazil [31–33]. In the Estonian population, HP prevalence is closely associated with the birth cohort [34, 35]: HP has become more rare among younger generations. In our bariatric surgery cohort, the detected 66% prevalence of HP is equivalent to that (56–69%) reported in previous studies on the same birth cohorts (1970–1990) of the general population in Estonia [35].

Because HP is associated with severe clinical sequels [19, 36–38], its eradication is indicated [13] and leads to regression of the inflammatory process in the gastric mucosa and significantly reduces the risk for its known complications at the population’s level. Indeed, the reported preoperative endoscopic findings (hiatal hernia, 16–25%; esophagitis, 13–30%) from geographic regions with low HP prevalence [13, 15, 30], as well as from the the high-prevalence regions [31–33], are consistent with similar morbidity in our cohort (Table 4). Chronic inflammation of the stomach mucosa was detected in 75% (49/65) and atrophy in 6.2% (4/65) of the patients. As expected, gastric diseases (gastritis, 65.1%; AG, 16.7%) are more frequent in regions [31, 33] with high HP prevalence, like Estonia, as compared with the low-prevalence regions (gastritis, 9.1–28%; AG 0.9%) [13, 15, 30].

Most of the patients in the NHS group had HP -related gastritis without atrophy. In such cases, gastroscopy is optional if the patient requests it [19]. Gastroscopy is mandatory only in the cases with suspected AG or in patients with sustained symptoms. In the NHS group, only four patients had moderate AGA which requires regular monitoring by endoscopy to disclose eventual progression and increased risk of gastric cancer [37, 39]. Of these four AGA cases, only one was clearly confirmed and another one was suspected on the basis of biomarker testing. In the other two cases, AGA was only confirmed with biopsy. In GastroPanel, the G-17 values were within normal limits, implicating that abundant G-cells were still present to sustain the normal G-17 output. Most likely, these cases represent patchy mucosal atrophy instead of a diffuse disease. It is not well established how such patchy atrophy behaves in the long run, and whether regular endoscopic monitoring is indicated or whether biomarker testing is sufficient. It is likely that the gastric mucosa in these patients can significantly recover after HP eradication, while inflammation symptoms diminish or disappear and the process of mucosal atrophy can be arrested, as reported earlier [24, 39, 40].

In our series, AGA detected by the biomarkers was rare, which has been shown before [41]. A recent meta-/analysis of the published GastroPanel literature confirmed that the test works better for detection of AGC (PgI, PgI/PgII ratio) than AGA (G-17). A simple explanation is that low G-17 levels can result from two distinct causes: AGA and high acid output [19, 20, 42]. No biomarker that is regulated by more than one trigger can be a highly specific indicator of only the other [20]. To make distinction between these two (AGA, high acid output), it is mandatory to test G-17 after protein stimulation (G-17 s). Failure to increase G17 s output implicates lack of G-cells and presence of AGA [20].

Another explanation for the rarity of AGA in our series could be the relatively young age of the patients. In fact, GastroPanel was not primarily designed for testing bariatric surgery patients but for diagnosis and screening of elderly patients with AG and for screening increased risk of gastric cancer [29]. However, bariatric surgery can be safely performed also in patients aged 60 years or more [43]. In this sub-group, the potential role of the gastric biomarker test can be particularly important as the incidence of atrophy and gastric cancer increases with age. Furthermore, using the biomarker test, we could easily diagnose almost all HP-infections and administer a timely treatment to diminish the risk of AG and gastric cancer.

There was also one false positive “panatrophy” (according to GP) in our series while histologically only superficial H.pylori related gastritis was confirmed. Rather, this fact could be related to technical issues.

In patients with AG, follow-up EGDS is still needed. Thus the gastric sleeve method (SG) would be preferable, because routine EGDS after bypass operation (GBP) is unfeasible. In large series of operated patients, however, practically no post-operative problems have been reported in the bypass group. Only a few case reports are available on postoperative cancer [44].

Regarding the use of the normal GastroPanel profile as a surrogate for healthy stomach (HS), 22/65 subjects were categorised into this group according to its criteria. Clinically, 20 of them were asymptomatic, had no history of abdominal complaints, and only 2 had reflux symptoms. On EGDS, only minor abnormalities were detected that were considered clinically insignificant: non-HP gastritis, mild or moderate degree esophagitis, or gastric mucosa erosions. It is clear that management of these disorders does not require a delay in elective surgery, nor is it a contraindication for operation [11]. In 4 cases, esophagitis (LA grade A/B) was found to be associated with hiatal hernia, and 2 of these reported reflux complaints. According to international consensus [17, 18], for patients with upper abdominal complaints, endoscopic investigation is indicated. In patients with symptomatic esophagitis, the recommended surgical procedure could be gastric bypass rather than gastric sleeve. Although the opinions on the use of gastric sleeve in esophagitis are controversial [45], the probability of complicated esophagitis has been shown to increase postoperatively [46]. Such cases respond poorly to medical treatment [47], despite the fact that a major portion of the gastric corpus is resected, which results in a significant reduction of parietal cell mass and a decline in acid output.

There is yet no unanimous agreement on the need and technical methods for simultaneous gastric sleeve and hiatal hernia repair, although most authors agree that posterior hiatus repair is necessary when hiatal hernia is diagnosed pre- or intraoperatively [47, 48]. It has been shown earlier that low basal G-17 levels in the general population are a marker of high basal acid output, which in turn predisposes to gastric acid reflux and esophagitis [42]. In this series, however, we failed to find correlation between esophagitis and low G17b levels, as only one out of the 4 patients in the HS group and 2/10 in the NHS group showed G-17b levels below the cut-off value. Although some studies have obtained results similar to ours [49, 50], there are also reports about such correlation between G-17 and esophagitis [51].

In the light of the above data, it is evident that in symptomatic esophagitis, endoscopy plays a role also in guiding the selection of the surgical method (i.e., preferring gastric bypass over gastric sleeve), which is crucial to ensure optimal treatment outcome. In our series, 3 asymptomatic patients in the HS group had, despite the normal marker profile, erosions in the stomach (antrum), with a Lanza score of less than 4 (i.e. not severe). Recently, some authors have reported gastric erosions in bariatric surgery patients [48] and others have reported them also in asymptomatic volunteers in population studies, more frequently in HP-negative than HP-positive subjects [40]. Although the cause of such erosions may be multifactorial, all 3 patients in our study took several medications known to damage the gastric mucosa. Yet the erosions seen in the HS group can be considered clinically insignificant: the patients were asymptomatic, and no complications like haemorrhage were found on EGDS. Accordingly, we cannot consider minor erosions in patients with the normal biomarker profile as an indication for changing treatment practices in such bariatric surgery patients.

Summing up for the HS group, our data demonstrate that the gastric biomarker test can definitely help select this particular patient group of asymptomatic patients (20/22 in this series) with minor but clinically non-significant gastric mucosa alterations for whom preoperative endoscopic investigation can be safely replaced by the non-invasive biomarker test. Endoscopy should only be reserved for symptomatic patients to confirm the diagnosis and opt for the surgical method, as has been pointed out earlier [17, 18].

Regarding the NHS patients, the rationale should be the same as for the HS patients: those with reflux complaints should undergo endoscopic investigation to confirm the diagnosis and to plan possible preoperative treatment. Endoscopic findings in bariatric surgery patients can be highly variable [52]. To avoid postoperative complications, including ulcer [52], it is important to evaluate the patients pre-operatively to detect (by using GastroPanel) [19] and eradicate HP infection. Although there were no cases of ulcer disease in our material, these steps are always important in this special group of patients. In the case of suspected peptic ulcer, EGDS is essential; the same applies to patients with a family history of gastric cancer.

The present results confirm that the normal biomarker profile in the GastroPanel test is an excellent surrogate for healthy stomach, and this non-invasive test could replace EGDS in the pre-operative management of bariatric surgery patients. Indeed, using the biomarker test, it could have been possible to avoid EGDS in 20/22 patients in the HS group, i.e., in 31% (20/65) of all bariatric patients in our cohort. These asymptomatic patients with the normal biomarker profile are at very low risk to develop a clinically significant disease in the gastric mucosa, including peptic ulcer and gastric cancer [19, 53].

## Conclusions

The present study demonstrates good correlation between serum biomarkers and gastric mucosal status in preoperative assessment of bariatric surgery patients. In asymptomatic patients with the normal biomarker profile, endoscopic investigation can be safely abandoned, which brings significant economic and resource-related benefits. The potential cost-effectiveness of this strategy would also be population and country specific. Given the key causative role of HP in gastric pathologies, one can anticipate that the lower is HP prevalence in the population, the higher is the proportion of patients in whom EGDS can be avoided. The full benefits of the non-invasive biomarker screening of bariatric surgery patients for EGDS (high-risk patients only) can only be established in larger cohorts, with participants from different populations and with different prevalences of HP-infection.

## Abbreviations

AG: Atrophic gastritis (mucosal atrophy)
AGA: Atrophic antrum gastritis
AGC: Atrophic gastritis of the corpus
AGP: Atrophic pan-gastritis (AG of the antrum and corpus)
BMI: Body mass index
EGDS: Esophagogastroduodenoscopy
G17 s: Stimulated amidated Gastrin 17
G17b: Basal (fasting) Gastrin 17
GBP: Gastric bypass
GERD: Gastroesophageal reflux disease
HP: *Helicobacter pylori*
HPAb: *Helicobacter pylori* antibodies
HS: Healthy stomach
LA: Los Angeles classification of esophagitis
NHS: Non-healthy stomach
PgI: Pepsinogen I
PgII: Pepsinogen II
SG: Sleeve gastrectomy
UGI: Upper gastrointestinal
USS: Updated Sidney System
